# Menstruation—Normal and Abnormal

**Published:** 1902-10

**Authors:** C. A. Button

**Affiliations:** Holland, N.Y.


### Menstruation—Normal and Abnormal.

By C. A. BUTTON, M.D., Holland, N.Y.

IT WAS lately stated by a correspondent in a medical journal that ovulation continued through gestation and lactation; the menstrual function has also been spoken of as a depurifying agent; and many cases have come under observation where abnormal menstruation has not been successfully treated and where malignant growths have passed beyond surgical aid, due to a failure to recognise the true condition. The normal process in woman is regular and painless, and its abnormal occurrence presents diagnostic features indicating various pathological conditions which, though often of minor importance, and sometimes of grave import, are too often lightly passed by, the physician attributing the trouble to age or some other cause without a careful investigation; hence this paper on menstruation, both normal and abnormal.

First, taking up normal menstruation, what is it and why does it occur? In all forms of life, both animal and vegetable, we find the same cycle—birth, propagation of the species, death. In both the animal and vegetable world a certain amount of growth and development is attained before the function of reproduction is established, at which time activity of the generative organs involuntarily begins. Thus the blade of corn grows without attempt at reproduction until of sufficient vitality to nourish and ripen its fruit, at which time its silk is developed and awaits impregnation by the pollen from the tassels; and if this occurs it goes on to maturity and reproduces its kind, thus propagating the species; but if the tassels of the field be clipped and the waiting silk fails to receive impregnation, then there is no further use for that which was designed and developed by nature to take a specific part in reproduction and it degenerates and is cast off. This is what occurs in some form in all of the animal and vegetable world, and is an analogue of that which causes the menstrual flow in woman. Each plant and animal has its own special time for fecundation and gestation.

Nature has designed that during a certain period varying with individuals, woman shall prepare for and nourish those elements which develop into a new being, thus propagating the species, and to that end a Graafian follicle is matured and its ovum discharged about every 28 days, and in anticipation of and preparatory for this event, the uterus undergoes those changes necessary to fit it to receive and nourish an impregnated ovum; the endometrium becomes many times thicker, soft, highly vascular and thrown into folds ready to receive, envelop and nourish it, the vitality of the entire organism being drawn upon and centered in this process; if impregnation fails to occur, diverted nutrition is again turned toward the upbuilding of the general system and the superfluous tissue of the uterus, for which there is now no use, undergoes degeneration, the eroded capillaries bleeding to a more or less extent, which, together with the ovarian discharge, all mixed with mucus is discharged, constituting the menstrual flow. Hence the menstrual function is not a depurative, but simply an elimination of waste tissue which nature designated to be used in propagating the species but, failing of an impregnated ovum, is of no further use and is cast off.

The question naturally arises, is ovulation the cause of menstruation? Some have cited cases of menstruation continuing after double ovariotomy to prove that it was not, but they fail to show that a supernumerary ovary did not exist; and the cases cited were operated upon after the menstrual function had become thoroughly established and which might continue for a time through influence of past ovulation over the organism.

The menstrual function does not concern the generative organs alone, but involves the entire being which sends extra vitality through its nervous and circulatory system to those organs at the expense of the balance of the tissues, and the ovary in developing its ovum, is the influential factor causing those changes to take place in other tissues preparing to receive it; hence the influence of ovulation over the organism induces those changes which result in menstruation when nature's plans for propagating the species are not fully met, and may thus be said to be the cause of normal menstruation. Ovulation can be readily observed in the domestic fowl, where gestation takes place outside of the body; though diverted by man to his use as food, the egg is designed by nature only for reproduction, and as soon as the fowl begins to sit upon her eggs, which is her period of gestation, her production of eggs ceases only to be resumed after her brood has been reared or otherwise disposed of. And it is the same in woman; when conception takes place ovulation ceases until after delivery, and usually through lactation if not prolonged beyond the usual time, although there are exceptional cases where ovulation begins soon after delivery, even where the mother is nursing her child.

Having considered the physiology of normal menstruation, now let us consider the pathology of abnormal menstruation. First, taking up amenorrhea, we will pass its physiological occurrence before puberty, during pregnancy and usually lactation, and after the menopause and take up its pathological occurrence, which may be due to either local, constitutional or accidental causes. Among the local causes may be absence of some of the organs, as ovaries or uterus, or atrophy, or disease of the same, or we might find a sealed cervix, either congenital or acquired, and in this latter case the woman might be retaining the menstrual flow, the uterus enlarging each month and in a way simulating pregnancy. Among the constitutional causes are chlorosis, other forms of anemia, general debility, chronic wasting diseases, especially tuberculosis, delayed development and the like, while shock, sudden chill or wetting of the feet is the usual accidental cause. Thus it will be seen that it is very important to locate the cause before beginning treatment, for while emmenagogues, hot foot and sitz baths, etc., are proper for accidental causes they are entirely out of place and poor practice if due to constitutional or local troubles. If mechanical, the obstruction must be removed; if constitutional, build the system up to a point where the vitality of the organism is amply sufficient to prepare for and nourish an impregnated ovum and emmenagogues will seldom be needed.

Second, dysmenorrhea is perhaps the most common menstrual trouble among women; a few come to the physician for treatment, while many suffer on trying only domestic remedies for temporary relief. This trouble is nearly always curable if correct diagnosis is made and proper treatment given. Sometimes the pain occurs before the flow, ceasing, however, when it begins; sometimes it continues during the entire course and occasionally it occurs both before and after the flow, being absent during the discharge. Among the causes are endometritis, congestion of the organs contiguous to the uterus, constricted cervix, sensitive nerve endings and a hypersensitive nervous system.

Here, again, diagnosis of the cause is the first and important point to determine, the routine administration of sedatives and narcotics, regardless of the cause is poor practice and usually gives unsatisfactory results as regards a permanent cure. An obstructed cervix requires such surgical treatment as the nature of the obstruction indicates. Congestion may be combatted with rest in bed, counter irritation, hot applications, cupping and similar remedies, together with the administration of uterine sedatives. Endometritis may be treated medicinally with intrauterine irrigations and applications, or surgically by curetting and packing. The medical treatment will be found effective and preferable in many ways for a large number of cases. If gonorrhea be a contributing cause it must receive appropriate attention; irrigation with protargol, beginning with ½ per cent. solution and gradually increasing the strength as the tolerance of the uterus will bear it up to 2 per cent. and perhaps sometimes to 5 per cent., will usually be found satisfactory in gonorrheal cases. For sensitive nerve endings or in any case where a uterine sedative and antispasmodic is required, elixir cramp bark comp., a teaspoonful in a wineglass of hot water, repeated in half an hour if necessary, will be found valuable.

If the courses are irregular and painful, correct any constitutional trouble present and beginning about ten days before the proper time for menstruation, give one-third of a teaspoonful liquor sedans in a little water every three hours until the flow begins, then give the elixir cramp bark comp. if required for pain during the course; repeat for a few months and a permanent cure will usually result, the courses being regular and painless.

Menorrhagia rarely occurs as a pathological condition requiring treatment, except when accompanied with metrorrhagia. The normal flow of women varies greatly both as to length of time and quantity, and if the courses are regular and painless, interference is not indicated unless the loss is sufficiently excessive to produce symptoms, as anemia, vertigo, prostration, and the like, in which case the patient should be put in the prone position and kept so for a prolonged period of time and the flow regulated, for which purpose frequent small doses of hydrastis, hamamelis (if a good article), tr. cinnamon, or ergot will be efficient; then pursuant to a permanent cure the cause should be sought after which may be found to be a debilitated system, an impoverished blood stream, a predisposition to hemophilia or other similar conditions. When the cause is located treatment will be self suggested.

We will now pass to that form of abnormal menstruation which results from pathological conditions, always serious and often very grave—conditions from which many a poor sufferer has lost her life which often might have been saved had they been recognised and treated in time. Many times the fault lies with the woman in not seeking advice early, but too often it is in the physician's attributing the trouble to age or some other cause without a thorough investigation to ascertain the true condition. I refer to metrorrhagia; this always indicates a pathological condition usually located in the uterus or its appendages, in the form of endometritis, displacements, lacerations, adenoid growths or malignant disease. Although most of these may appear at any age, yet some of them are more liable to occur during certain periods in life than at other times; hence age is an aid to diagnosis; thus in a young woman we would look for endometritis and displacements, in the child-bearing period we would look for endometritis, displacements, lacerations, fibroids and polypi; while near the menopause it is very likely to be fibroids, polypi or malignant disease, and after the menopause, malignant disease is most often the trouble. Metrorrhagia occurring near the menopause should never be attributed to the climacteric, but an examination always made, submitting scraping of tissue to the microscope if necessary; for malignant disease if present and recognised early may often be eradicated by surgery, when left till other tissues are invaded, it may be too late and the case hopeless.

Polypoid growths should be removed, but with interstitial fibroids it depends on conditions present whether surgical interference is advisable or not; if the patient is near the climacteric, the menopause favors their absorption, and if there is not uncontrolable hemorrhage, pressure, or other symptoms of sufficient magnitude to call for immediate action, it is better to wait, alternating terms of ergot with hydrastis, in small doses, keeping watch of the tumor to detect softening and ascertain as to its change in size; if it ceases to grow, its absorption may be expected, but if it continues to grow rapidly, surgical interference will doubtless be required. Very many, however, will cease growing and be absorbed. In malignant disease, if recognised early, surgery may add many years to the patient's life; hence I repeat, in metrorrhagia always make a thorough examination of the uterus and all of the uterine appendages in an aseptic manner.

I have attempted in this paper to give hints which may lead to the mitigation of suffering, or induce more diligent and thorough investigation as to the cause of menstrual troubles, especially metrorrhagia, when occurring near or after the menopause, to the end that lives may be saved.

